# Host Diet and Species Interact to Shape the Bacterial and Fungal Microbiome in the Regurgitant of Four *Spodoptera* Species

**DOI:** 10.1007/s00248-025-02582-5

**Published:** 2025-07-22

**Authors:** Maximilien A. C. Adam, Guillaume Cailleau, Pilar Junier, Betty Benrey

**Affiliations:** 1 Laboratory of Evolutionary Entomology, Institute of Biology, University of Neuchâtel, Neuchâtel, Switzerland; 2 Laboratory of Microbiology, Institute of Biology, University of Neuchâtel, Neuchâtel, Switzerland; 3 Present Address: CIRAD, UMR CBGP, 34398 Montpellier, France; 4 Department of Entomology, The Pennsylvania State University, University Park, PA 16802 USA; 5 State Key Laboratory of Cotton Biology, School of Life Sciences, Henan University, Kaifeng, China

**Keywords:** Microbial community, 16S rDNA sequencing, ITS rDNA sequencing, Gut bacteria, Gut fungi, Microbiota, Foregut microbiome

## Abstract

The gut microbiome of Lepidopteran insects is highly dynamic, influenced by both host diet and phylogeny. While microbial communities are thought to facilitate host adaptation to diverse diets and environments, the existence of a core microbiome shared among closely related herbivores remains largely untested. In this study, we examined the microbial communities in the regurgitant of four S*podoptera* species (*S. exigua*, *S. frugiperda*, *S. latifascia*, and *S. littoralis*) across different diets (artificial diet, cotton, maize, and squash). Using a high-throughput sequencing, we characterized bacterial and fungal community composition and diversity. Bacterial communities were shaped by both diet and host species, indicating species-specific bacterial selection. In contrast, fungal communities were exclusively structured by diet, with lower diversity and dominance of a few key taxa. Notably, no operational taxonomic units were consistently shared across all species or diets, challenging the concept of a conserved core microbiome in these generalist herbivores. Understanding how microbial communities shape generalist herbivores’ ability to feed on diverse plants may offer potential strategies for microbiome-based pest management.

**Supplementary Information:**

The online version contains supplementary material available at 10.1007/s00248-025-02582-5.

## Introduction

Herbivorous insects often associate with gut microorganisms, including bacteria and fungi, which influence various aspects of their biology [1, 2]. Among the factors influencing these interactions, diet plays an important role [3]. This is particularly evident in lepidopteran larvae, whose gut microbiome is characterized as simple and highly variable [4]. Such variability is attributed to continuous moulting [5], the absence of specialized gut compartments, and the alkaline conditions of their digestive system [6]. As a result, lepidopteran gut microbiomes are largely determined by environmental factors and diet, showing variation both among and within species [7]. In addition to deterministic factors, microbial acquisition can also be strongly influenced by stochastic processes, such as random exposure to microbes in soil or plant surface, which contributes to inter-individual variability [8]. The relative contribution of stochastic processes varies across insect species [9]. Despite this variability, gut bacteria contribute to key functions, including digestion [10], use of suboptimal diets [11], pathogen resistance [12], insecticide resistance [13] and manipulation of plant defences [14]. While some studies suggest that caterpillars lack a resident gut microbiome [15], others have shown that some bacteria can colonize and persist in their gut [5, 11, 16]. It has also been proposed that a subset of functionally important bacteria may form a core microbiome shared across lepidopteran species [17, 18]. For such core microbiome to exist, either vertical transmission or constant horizontal acquisition of microbes would be required [7]. However, only a limited number of systems have tested this hypothesis, yielding inconsistent results [4, 7, 19, 20]. Thus, whether closely related herbivorous insects harbour a core gut microbiome remains unresolved.

The superfamily Noctuoidea is the largest and more diverse group within Lepidoptera, including many agricultural pest species [21, 22]. Within this group, the genus *Spodoptera* (Lepidoptera: Noctuidae) comprises several economically important species, including *S. frugiperda*, *S. littoralis*, *S. exigua* and *S. latifascia* [21, 23]. S*podoptera frugiperda* and *S. exigua* have invaded multiple continents, and all four species are highly polyphagous [21, 24]. Understanding the factors enabling *Spodoptera* species to exploit diverse host plants is crucial for pest management strategies that target the insect microbiome through biochemicals or foreign microbes [25]. Gut microbes are thought to play a key role in this adaptability by facilitating host shifts and supporting polyphagous diets [7].

Over the past decade, several studies have characterized the gut bacterial composition of *S. frugiperda* [26–31], *S. littoralis* [4, 5, 32, 33] and *S. exigua* [34–38]. However, the gut microbiome of *S. latifascia* remains unexplored, and no comparative studies have examined gut microbiota across multiple *Spodoptera* species. This limits our understanding on how microbial communities contribute to the genus’ dietary adaptability. In contrast to bacteria, the fungal microbiota of *Spodoptera* caterpillars has received less attention [39–41], despite evidence from other lepidopteran insects showing that fungi are common in their guts [42–45]. Like bacteria, gut fungi may contribute to food detoxification and nutrient supply [46, 47], but it is unclear whether fungal microbiota is as simple and variable as bacterial communities in lepidopteran species.

Given the high degree of dietary overlap and similar gut physiology among *Spodoptera* species, it is plausible that they may harbour a shared set of microbial associates. We hypothesized that this putative core microbiome shared among *Spodoptera* species facilitates their adaptation to diverse host plants. To test this, we examined 1) whether microbial community compositions in the regurgitant of four *Spodoptera* species (*S. exigua*, *S. frugiperda*, *S. latifascia*, and *S. littoralis*) exhibit similarities, and 2) how these communities are influenced by different diets (artificial diet, cotton, maize and squash). Despite extensive research on lepidopteran microbiota, most studies focus on single species or specific environmental variables, limiting cross-species comparisons within ecologically important groups. Our study fills this gap by providing a comparative analysis of gut microbiota across multiple *Spodoptera* species under standardized conditions. These insights can potentially inform pest management strategies aimed at suppressing or replacing a symbiont by another microbe [25] and contribute to broader ecological and evolutionary perspectives on host-microbe interactions.

## Materials and Methods

### Artificial Diet and Plants

The artificial diet used in this experiment (“beet armyworm diet”) was obtained from Bio-Serv (U.S.A., diet composition described in [48]). Cotton, maize and squash plants were grown from seeds. Cotton seeds were collected on feral plants near Puerto Escondido (Oaxaca, Mexico). Maize and squash seeds were obtained from Delley Semences et Plantes SA (Switzerland) and Zollinger (Bio Sàrl, Switzerland), respectively. These diets were selected to reflect the natural feeding habits of *Spodoptera* species, with artificial diet serving as a standardized control and the plant species representing common agricultural crops frequently consumed by these pests. Seeds were sown in a commercial soil (Aussaaterde, Ricoter, Switzerland) in plastic pots (11 cm height, 4 cm diameter) and plants were grown in a climatised greenhouse with additional lights (light 12 h/dark 12 h (L:D)).

### Insects

*Spodoptera littoralis* is native to Africa and has spread to Southern Europe and the Middle East, where it feeds on crops such as wheat, maize, cotton and rice [49]. *S. exigua*, originally from Asia, has become a globally distributed pest that targets major crops such as cotton, soybean, potato and sugar beet [50]. *S. frugiperda* is an invasive species native to the Americas, primarily attacking maize and other cereals [24]. *S. latifascia* is a polyphagous insect native to Mexico and Central America that commonly feeds on maize, bean, cotton and potato [51]. Eggs of *S. littoralis* and *S. exigua* were obtained from Syngenta (Stein, Switzerland) and Entomos AG (Grossdietwil, Switzerland), respectively. *S. frugiperda* and *S. latifascia* were initially collected near Puerto Escondido (Oaxaca, Mexico; 15°55′33.3″N, 97°09′03.0″W) and reared for several generations on a chickpea flour-based artificial diet [52] under controlled conditions (26 °C, 60% relative humidity (r.h.) and light (12:12 h L:D). New individuals were added to the colony each year to maintain genetic diversity.

### Experimental Design

A total of 140 seeds from each plant species were individually sown in plastic pots and placed in a greenhouse. After one month, three egg batches from each *Spodoptera* species were placed in separate Petri dishes, in a climate-controlled room (24 ± 2 °C, 40 ± 5% r.h.), under light benches (16:8 h L:D, approx. 150 lmol m^−2^ s^−1^), until the end of the experiment. All eggs hatched on the same day, except for *S. latifascia*, which hatched one day later. On the day of hatching, first instar caterpillars were randomly selected from the three egg batches and transferred to 16 rearing trays (one tray with 32 cells per diet treatment, Frontier Agricultural Sciences, USA). Two caterpillars were placed in each cell to compensate for the high mortality of first instars. After one week, if both caterpillars survived, one was removed. Caterpillars were fed one of the four diet treatments: small cubes (1 cm^3^) of artificial diet or cut pieces of cotton, maize or squash leaves (2 cm^2^). Food was replaced as needed, at least twice per week. Once caterpillars reached the fourth instar, regurgitant was collected daily over three consecutive days. Regurgitant originates from the foregut of caterpillars, and its microbiome differs significantly from that of the midgut [29]. It often comes in contact with plant tissue, and the microbes it contains can affect plant responses to herbivory [53]. To collect regurgitant, larvae were gently poked by hand and placed in a clean Petri dish. Regurgitant was then extracted using a 20–200 μl pipet and transferred into 1.5 mL Eppendorf tubes stored at −80 °C until further analyses. Each Eppendorf tube contained pooled regurgitant from 10–11 individuals. In total, we obtained three regurgitant solutions per combination of *Spodoptera* species and diet (number of replicates per treatment combination: n = 3). To prevent cross-contamination, gloves, Petri dishes and pipet tips were changed between treatments, and the work surface was disinfected with alcohol after each procedure.

### DNA Extraction and Sequencing

DNA was extracted using the FastDNA Spin Kit for soil (MP Biomedicals), following the standard protocol provided with the kit. DNA quantification was performed using the Qubit® dsDNA HS Assay Kit on a Qubit® 2.0 Fluorometer (Invitrogen, Carlsbad, CA, USA). Purified DNA extracts were sent to Fasteris (Geneva, Switzerland) for bacterial 16S rDNA and fungal internal transcribed spacer (ITS) amplicon sequencing using an Illumina MiSeq platform (Illumina, San Diego, USA), generating 250 bp paired-end reads. For the 16S rDNA, the V3–V4 region was amplified using the universal primers Bakt_341F (5’-CCT ACG GGN GGC WGC AG-3’) and Bakt_805R (5’-GAC TAC HVG GGT ATC TAA TCC-3’) [54]. The primers ITS3_KYO2 (5’-GAT GAA GAA CGY AGY RAA-3’) and ITS4 (5’-TCC TCC GCT TAT TGA TAT GC-3’) [55] were used for the amplification of the ITS2 region. DNA extraction controls were unfortunately not included in the analysis.

### Bioinformatic Analysis

Demultiplexed and trimmed sequence reads provided by Fasteris were processed using QIIME2 [56] with DADA2 [57] for the denoising step. For 16S reads, sequences were truncated to optimized lengths based on quality scores, with forward and reverse reads set to 264 and 216 bases, respectively. This resulted in a total composite length of 480 bases for unjoined sequences. The truncated reads allowed for the joining of denoised paired-end sequences with at least 12 identical bases, yielding full-length denoised sequences of 468 bases. The resulting Amplicon Sequence Variants (ASVs) were taxonomically classified using QIIME2’s VSEARCH-based consensus taxonomy classifier [58] with the SILVA database, release 138 [59]. A refined version of the SILVA database, curated using the RESCRIPt QIIME2 plugin [60] and provided by the QIIME2 team, was employed for this purpose. For ITS reads, sequences were truncated to optimized lengths based on quality scores, with forward and reverse reads set to 264 and 216 bases, respectively, resulting in a composite length of 480 bases for unjoined sequences. These truncated reads allowed the joining of denoised paired-end sequences with at least 12 identical overlapping bases, producing full-length denoised sequences of 468 bases. The sequences were grouped into ASVs, which were subsequently taxonomically classified using QIIME2’s VSEARCH-based consensus taxonomy classifier with the UNITE database (version 8.2, release date February 4, 2020 [61]). The data set was decontaminated using the microDecon R package [62, 63] based on a PCR control sample [64]. We applied a 0.1% rarefaction threshold [42, 65, 66] and rarefied the sequences to 2000 per sample to correct for uneven sequence numbers. This rarefaction depth represented the lowest number of high-quality sequences retained across all samples after quality filtering. This approach allowed us to include all samples in the analysis while maintaining consistency in sequencing depth.

### Statistical Analyses

All statistical analyses were performed using R version 4.4.1 [67]. Rarefaction curves, illustrating the relationship between sequencing depth and the number of observed Amplicon Sequence Variants (ASVs), were generated using the ‘Phyloseq’ package in R. To visualize bacterial and fungal community composition, we used the ‘Vegan’ package [68] which generates non-metric multidimensional scaling (NMDS) using the Bray–Curtis coefficient as the distance measure. The Bray–Curtis coefficient was calculated using Hellinger-transformed relative abundance data of observed bacteria and fungi in each sample. The homogeneity of variances among diet and species was tested using the R function ‘betadisper’. Differences in microbial communities across diet treatments and *Spodoptera* species were tested using permutational multivariate analysis of variance (PERMANOVA), via the *adonis* function in ‘Vegan’ [69]. The model included diet, species and their interactions as fixed factors. Significance was determined through 1,000 permutations, followed by posthoc pairwise comparisons (*pairwise.adonis*) when significant effects were detected.

Shannon diversity indices were calculated using the ‘Phyloseq’ package in R, and differences in diversity were analyzed using ‘lme4’ [70], ‘nlme’ [71] and ‘emmeans’ [72] packages in R. Residuals were visually inspected for normality and homogeneity. Separate linear models were used to analyse Shannon and Pielou diversity for bacteria and fungi, while a linear mixed model was applied to assess bacterial and fungal diversity jointly. In the mixed model, diet and *Spodoptera* species were treated as random factors, while in the linear models, they were fixed factors. When significant differences (< 0.05) were detected, posthoc Tukey’s honestly significant difference (HSD) tests were conducted for pairwise comparisons.

## Results

After quality filtering and rarefying, we retained a total of 4648 bacterial ASVs and 843 fungal ASVs for further analyses. Rarefaction curves approached saturation, indicating that our sequencing depth adequately captured microbial diversity (Fig. S1). The three regurgitant samples collected from *Spodoptera littoralis* fed on cotton did not yield any reads and were excluded from the analysis. Due to high larval mortality, the following treatment combinations were excluded from the analyses: *Spodoptera exigua* fed on maize and *Spodoptera latifascia* fed on squash. The remaining treatments included in the analyses were: *S. exigua* fed on artificial diet, cotton, and squash; *S. frugiperda* fed on artificial diet, cotton, maize and squash; *S. latifascia* fed on artificial diet, cotton and maize; *S. littoralis* fed on artificial diet, maize and squash (Table S1).

### NMDS

**Fig. 1:**
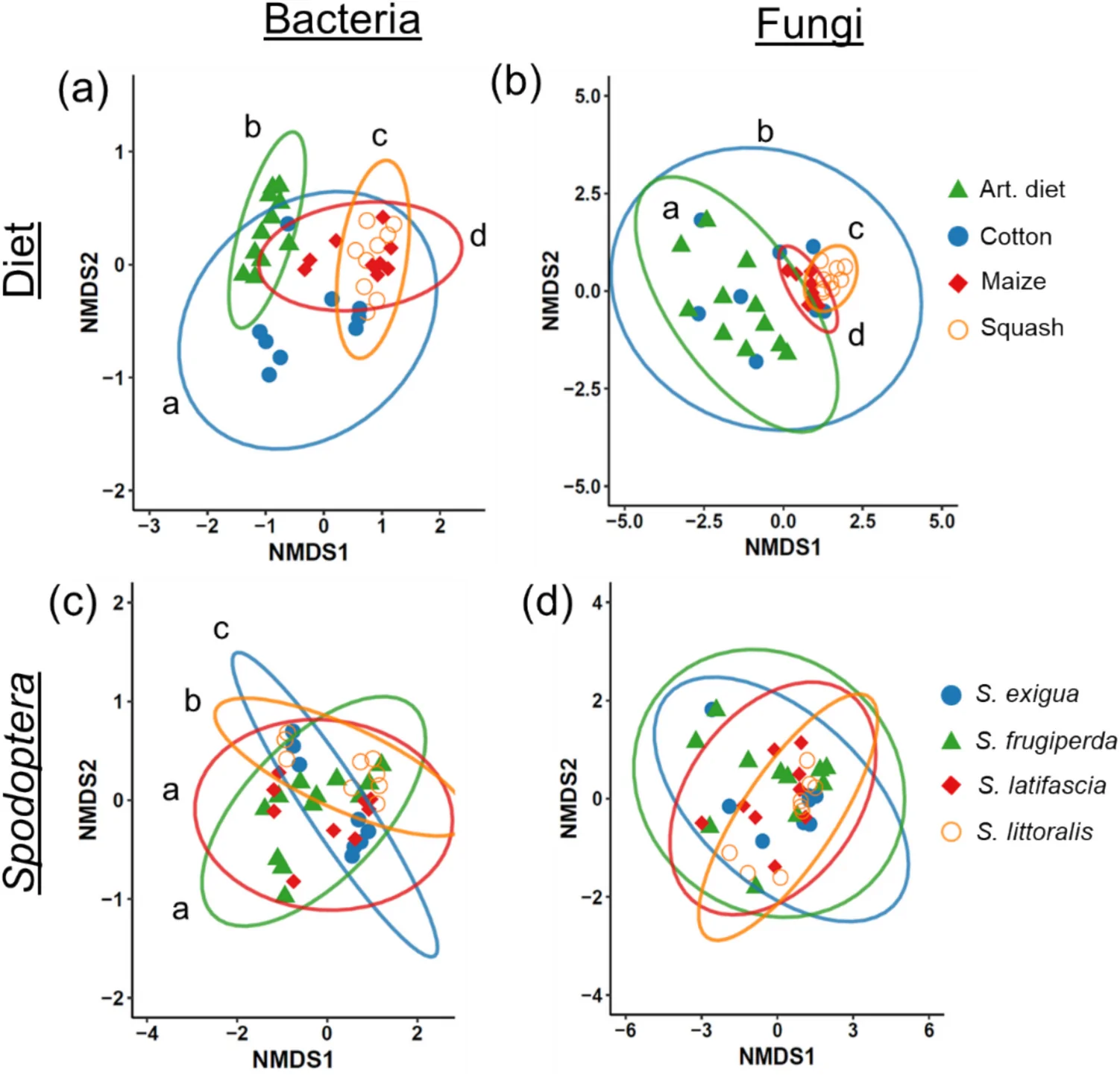
Non-metric multidimensional scaling (NMDS) ordination plots based on Bray–Curtis distances of Hellinger-transformed relative abundances of *Spodoptera* regurgitant microbial communities. (**a**) Bacterial and (**b**) fungal communities in *Spodoptera* regurgitant grouped by diet. (**c**) Bacterial and (**d**) fungal communities in *Spodoptera* regurgitant grouped by species. Each point represents one regurgitant sample. Each treatment combination of *Spodoptera* species and diet was replicated three times (n = 3), with each replicate consisting of pooled regurgitate collected from 10–11 individuals. Ellipses represent 95% confidence intervals around the group mean (diet or species). Different letters indicate significant differences among treatments (pairwise posthoc analysis). NMDS stress values (goodness of fit): bacteria = 0.118; fungi = 0.121

Analysis of multivariate dispersion using the betadisper function revealed a significant effect of diet treatment (Fig. S2 and S3), whereas no significant differences in dispersion were found among *Spodoptera* species. Diet had a significant effect on both bacterial (*F* = 9.925, *df* = 3, *p* < 0.001) and fungal (*F* = 5.037, *df* = 3, *p* < 0.001) community compositions in caterpillar regurgitant (Fig. 1a, b). Posthoc pairwise analyses revealed that each diet treatment influenced bacterial and fungal community compositions differently (Fig. 1a, b). The *Spodoptera* species also had a smaller but significant effect on bacterial community composition (*F* = 2.704, *df* = 3, *p* < 0.001), with the exception of *S. frugiperda* and *S. latifascia* which showed no significant differences (posthoc pairwise analysis: *F* = 1.186, *df* = 1, *p* = 0.260) (Fig. 1c). In contrast, fungal community composition was consistent across the four *Spodoptera* species (*F* = 1.328, *df* = 3, *p* = 0.120) (Fig. 1d). A significant interaction between diet and species was observed for bacterial community composition (*F* = 2.245, *df* = 6, *p* < 0.001) (Fig. S4a), while no significant interaction was found for fungi (*F* = 1.342, *df* = 6, *p* = 0.080) (Fig. S4b).

### Community Composition

**Fig. 2:**
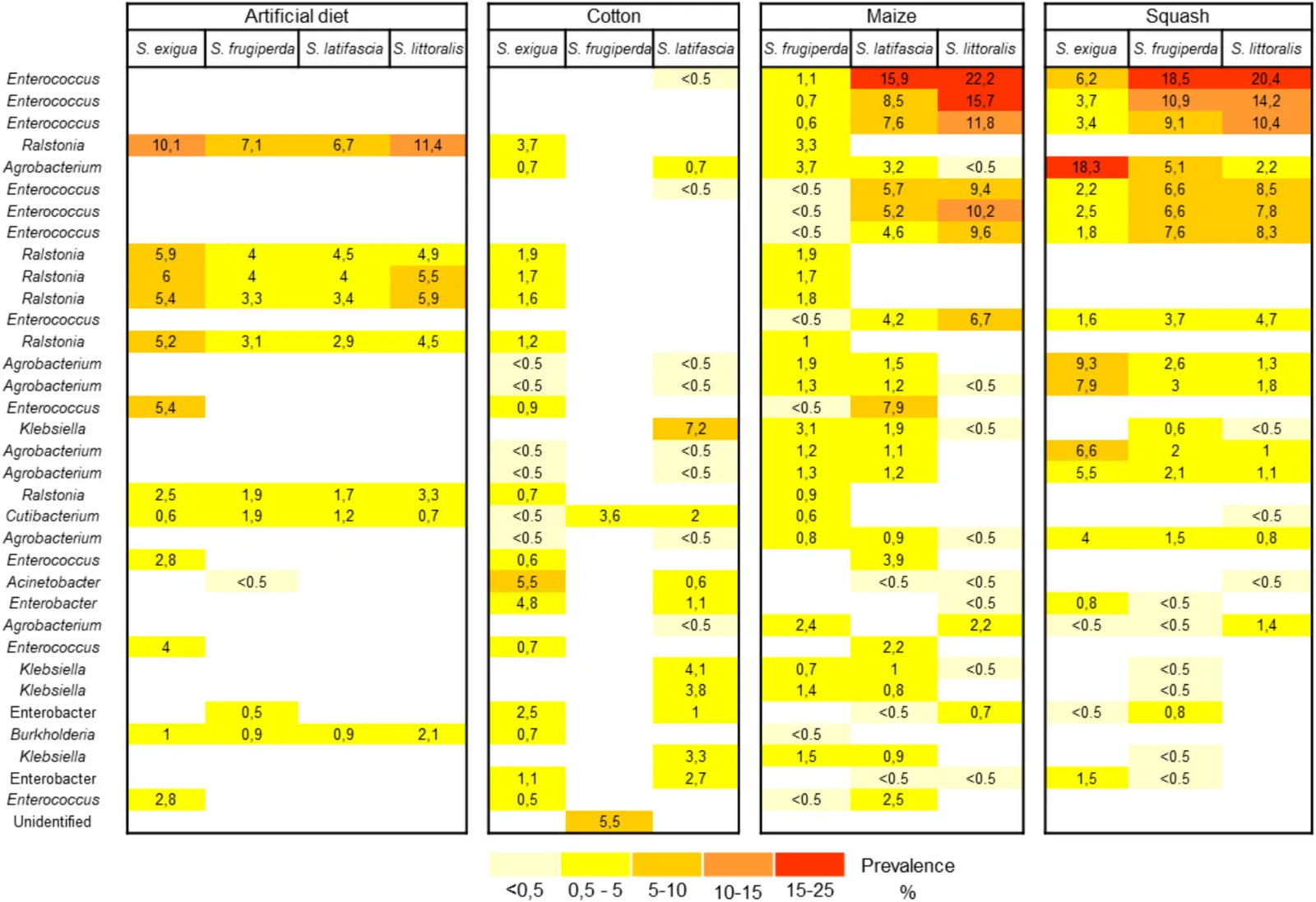
Bacterial community profiles of regurgitant collected from caterpillars of four species of *Spodoptera* (*S. exigua*, *S. frugiperda*, *S. latifascia*, and *S. littoralis*) fed on four different diets (artificial diet, cotton, maize, or squash plants). The bacterial taxa shown represent the 35 most prevalent ASVs identified at the genus level (full list is shown in Table S2). Colours represent the average prevalence (%) of bacterial taxa within each group, with white indicating absence

The bacterial communities in *Spodoptera* regurgitant were primarily dominated by members of the phyla Firmicutes and Proteobacteria. Notably, *Enterococcus* species were highly abundant in caterpillars fed on maize and squash, but were absent in those fed on artificial diet or cotton (Fig. 2). We also observed a higher number of members of the genus *Ralstonia* in the regurgitant of caterpillars fed with artificial diet. Overall, bacterial community profiles were largely consistent among *Spodoptera* species within the same diet, except for *S. frugiperda* on cotton, which showed a distinct bacterial composition. We found no taxa consistently present across all *Spodoptera* species (i.e. no taxon was detected in every sample). Even within each *Spodoptera* species fed with different diets, no shared microbes were detected. This absence was confirmed both before and after filtering and rarefaction.

**Fig. 3:**
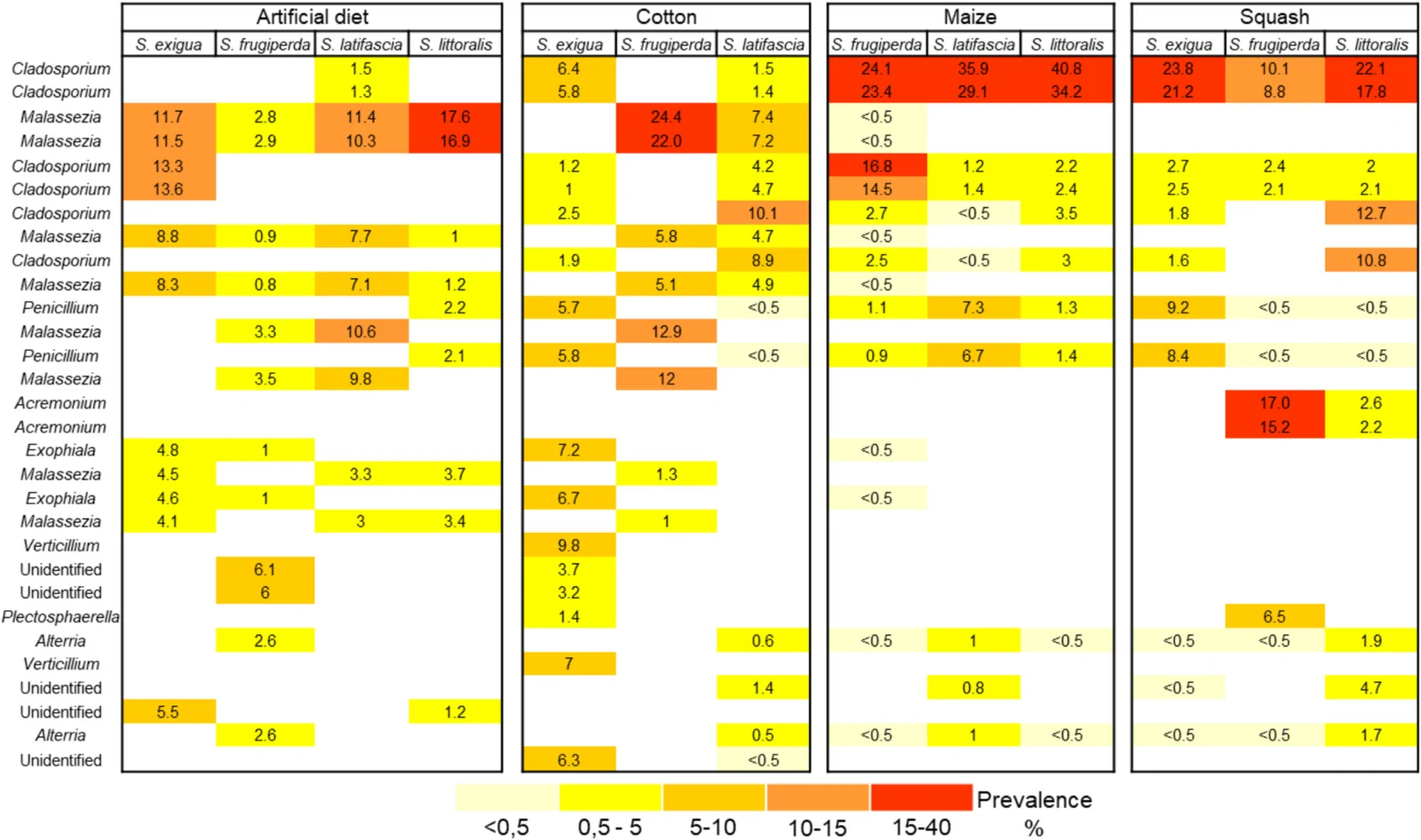
Fungal community profiles of regurgitant collected from caterpillars of four species of *Spodoptera* (*S. exigua*, *S. frugiperda*, *S. latifascia*, and *S. littoralis*) fed on four different diets (artificial diet, cotton, maize, or squash plants). The fungal taxa shown represent the 30 most prevalent ASVs identified at the genus level (full list is shown in Table S3). Colours represent the average prevalence (%) of fungal taxa within each group, with white indicating absence

Fungi identified in the regurgitant belonged primarily to the phyla Ascomycota and Basidiomycota. *Cladosporium* species were the most abundant fungi in caterpillars fed on maize or squash, whereas *Malassezia* species dominated in those fed on artificial diet and cotton (Fig. 3). Similar to bacteria, we found no fungal taxa consistently present across all *Spodoptera* species. Fungal community composition was primarily shaped by diet rather than *Spodoptera* species, although this pattern was less clear in caterpillars fed on cotton.

### Diversity

**Fig. 4:**
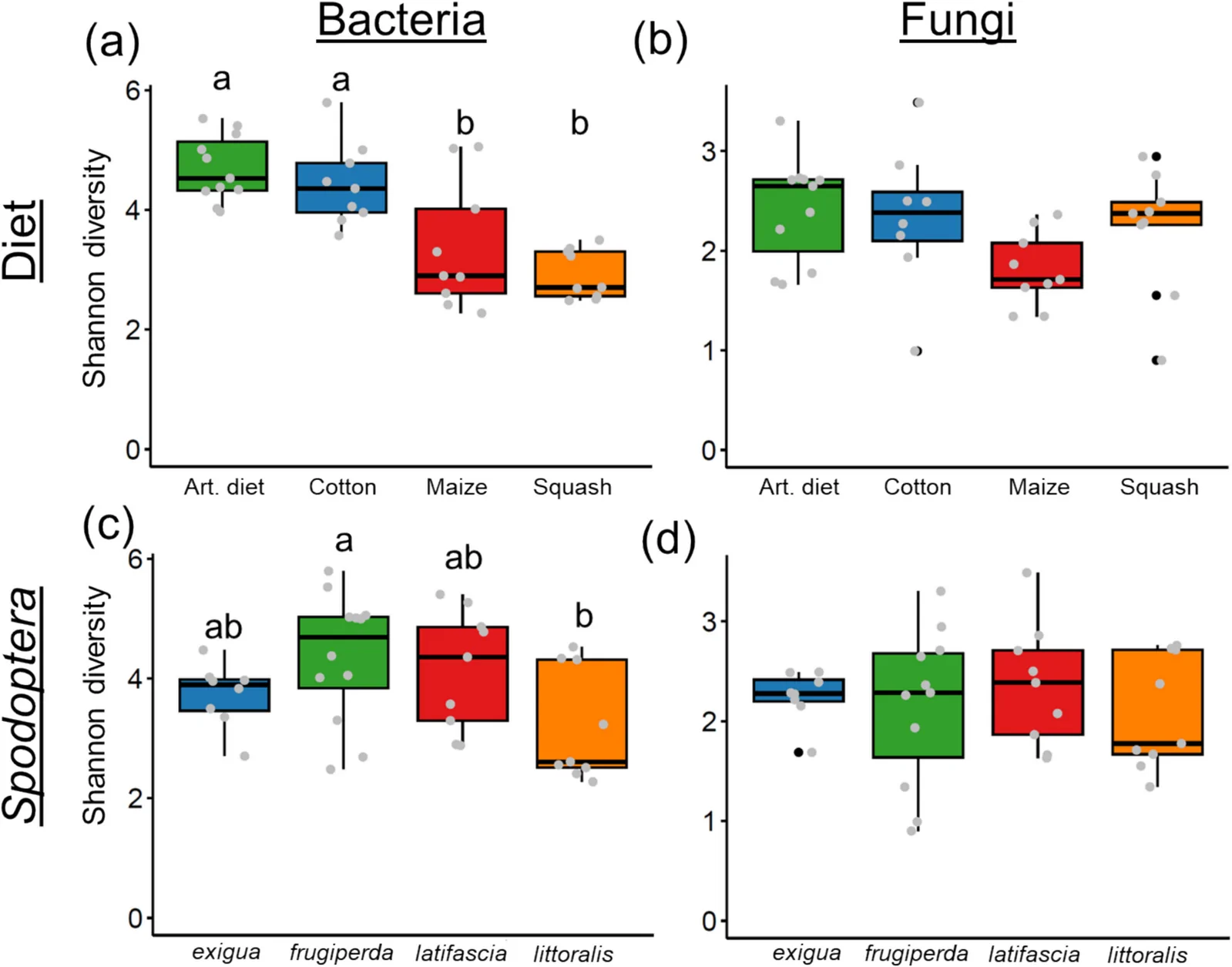
Shannon diversity indices of bacteria (**a**, **c**) and fungi (**b**, **d**) in *Spodoptera* regurgitant, grouped by diet (**a**, b) and species (**c**, **d**). Each treatment combination of *Spodoptera* species and diet was replicated three times (n = 3), with each replicate consisting of pooled regurgitate collected from 10–11 individuals. Grey dots represent individual raw data points (jittered for clarity), while black dots represent outliers included in the analyses. Different letters indicate significant differences among treatments (*p* < 0.05, Tukey’s HSD)

**Fig. 5:**
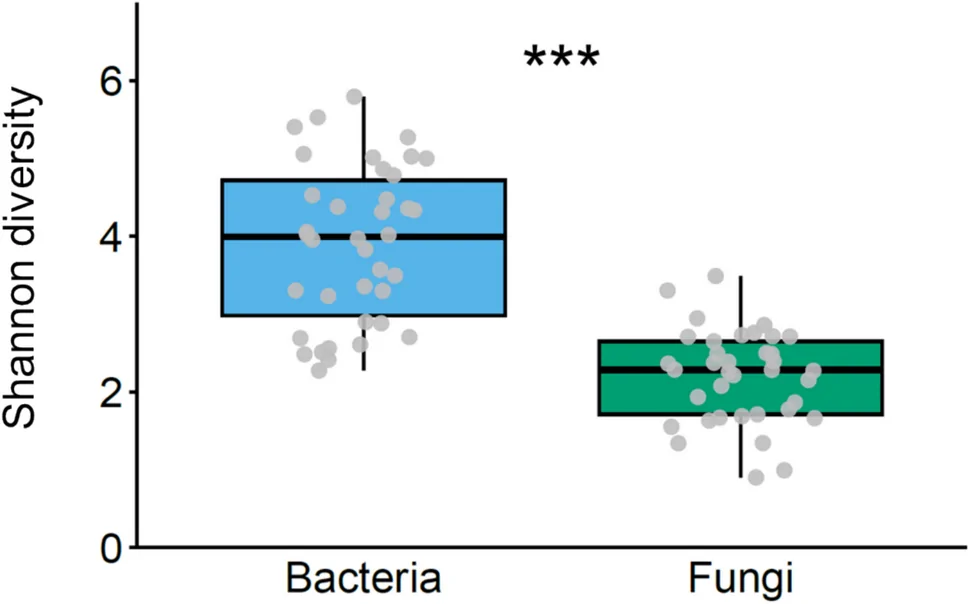
Shannon diversity indices of bacteria and fungi in the regurgitant of four *Spodoptera* species (*S. exigua*, *S. frugiperda*, *S. latifascia* and *S. littoralis*) fed on four different diets (artificial diet, cotton, maize and squash). Each treatment combination of *Spodoptera* species and diet was replicated three times (n = 3), with each replicate consisting of pooled regurgitate collected from 10–11 individuals. Grey dots represent individual data points (jittered for clarity), and the black dot indicates an outlier included in the analysis. Asteriks indicate significant differences between bacterial and fungal diversity (*p* < 0.001, Tukey’s HSD)

Shannon diversity indices for bacterial ASVs varied significantly according to caterpillar diet (*F* = 28.559, *df* = 3, *p* < 0.001) (Fig. 4a). Posthoc analyses indicated no significant differences between caterpillars fed on artificial diet and cotton (*t* = 0.269, *df* = 34, *p* = 0.836) or between those fed on maize and squash (*t* = 1.366, *df* = 34, *p* = 0.529). *Spodoptera* species also significantly influenced Shannon diversity indices (*F* = 8.729, *df* = 3, *p* < 0.001), with *S. frugiperda* exhibiting higher diversity than *S. littoralis* (posthoc: *t* = 2.818, *df* = 34, *p* = 0.038) (Fig. 4c). A significant interaction between diet and species was observed for bacterial ASVs (*F* = 5.176, *df* = 6, *p* = 0.001) (Fig. S5). In contrast, neither diet (*F* = 2.527, *df* = 3, *p* = 0.081) nor species (*F* = 0.490, *df* = 3, *p* = 0.692) significantly affected Shannon diversity indices for fungal ASVs (Fig. 4b, d; Fig. S6). The same results were found for Pielou diversity indices (Fig. S7). Overall, Shannon bacterial diversity in *Spodoptera* regurgitant was significantly higher than fungal diversity (*F* = 113.600, *df* = 1, *p* < 0.001) (Fig. 5).

## Discussion

We hypothesized that *Spodoptera* species might share a core microbiome that facilitates their adaptation to diverse host plants. To test this, we examined microbial community compositions in the regurgitant of four *Spodoptera* species (*S. exigua*, *S. frugiperda*, *S. latifascia*, and *S. littoralis*), and assessed the influence of diet. Our results revealed that diet strongly shaped both bacterial and fungal communities in *Spodoptera* regurgitant. While bacterial communities differed significantly among species, fungal communities remained largely consistent. Moreover, diet effects on microbial composition varied by *Spodoptera* species, suggesting species-specific microbial selection mechanisms. However, no bacterial or fungal ASV was consistently present across all treatments, indicating the absence of core microbiome shared by *Spodoptera* species. Overall, bacterial and fungal communities were shaped more by diet than by species, and fungal communities were less diverse than bacterial communities.

### Diet as a Driver of Microbial Composition

The strong effects of diet on microbial communities aligns with previous research on lepidopteran larvae, where gut bacterial community composition is often diet-dependent [18].

For example, host plant species or genotype significantly influenced gut bacterial microbiota in *S. frugiperda* [26, 28–31, 41], *S. exigua* [36–38] and *S. littoralis* [4]. Similarly, Yuning et al. [41] found that fungal communities in *S. frugiperda* midgut varied with host plant species (*Brassica campestris* vs *B. oleracea*). To our knowledge, no studies have examined microbial community compositions in *S. latifiacia* or fungal communities in *S. exigua* and *S. latifascia*. Diet-driven shifts in microbial communities have important implications for plant–insect interactions. Microbial communities can mediate digestion [11], pesticide resistance [13], plant defense manipulation [14], and even attraction of hyperparasitoids [62]. Understanding these dynamics may help identify symbionts essential for host adaptability or potential microbial targets for biological control strategies [25].

### Absence of a Core Microbiome

Previous studies suggested that *Spodoptera* species share a core microbiome [4, 7, 18], potentially including vertically transmitted microbes providing essential functions. For instance, *Enterococcus mundtii* was found in the second generation of *S. littoralis* after ingestion [5]. Similarly, geographically distant *S. frugiperda* populations harboured similar gut bacterial communities [20]. In contrast, our findings revealed distinct bacterial compositions in *Spodoptera* regurgitant, except for similarities between *S. frugiperda* and *S. latifascia*, possibly due to their shared evolutionary history in southern Mexico’s, milpa agroecosystem [73, 74]. Previous research also showed that gut bacterial communities of *S. frugiperda* [31] and *S. exigua* [38] differ when collected from the same plant species but different locations. Unlike bacteria, fungal communities were similar across species. However, as no fungal ASV was consistently shared among species when fed different diets, *Spodoptera* species likely do not share a core mycobiome. Research on tree caterpillars similarly suggests that host species play a minor role in shaping fungal communities [44].

### Diet-Species Interactions Shape Bacterial Communities

The interaction between diet and species influenced both bacterial composition and diversity. For example, *S. frugiperda* showed higher bacterial diversity than *S. latifascia* and *S. littoralis* when fed maize, which could reflect its specialization on this crop [75]. However, it should be noted that a diverse gut microbiome can also be associated with a large diet breath [76, 77], while other studies found no relationship [78, 79]. Lower bacterial diversity in maize- and squash-fed caterpillars, may be attributed to the dominance of *Enterococcus* species, which were absent in caterpillars fed artificial diet or cotton. *Enterococcus* species are particularly relevant as they may contribute to pesticide tolerance [80]. These findings suggest that caterpillars selectively retain specific bacteria from their diet. For example, *E. mundtii* in *S. littoralis* produces bactericidal compounds that selectively eliminate foreign bacteria [1, 81]. Additionally, microbial selection may occur due to the highly alkaline conditions of the digestive system [6].

### The Importance of Studying Fungal Communities

Consistent with previous studies [41, 42], fungal diversity was lower than bacterial diversity, with fungal communities dominated by a few highly prevalent taxa, such as *Cladosporium* and *Malassezia*. This pattern may result from antifungal activity mediated by gut-associated bacteria [82]. Another reason for the restricted fungal diversity could be a bias of amplification caused by the primers selected. Primer specificity, or the lack of primer universality has been a persistent issue that has not fully been addressed for biodiversity studies in Fungi using the Illumina platform [83]. The forward primer ITS3_KYO2 was designed to improve the coverage of previous primers for the 5.8S rRNA gene. In contrast, the primer ITS4 was already shown to have a very good coverage for the large ribosomal subunit. The combination, of both primers offered the best compromise for the amplification of the ITS2 region and sufficiently informative in the context of ecological and microbiological studies [55]. Unfortunately, this primer pair was not included in a more recent comparison of primer performance [84] and therefore it is difficult to determine their level of universality. *Cladosporium* species, commonly found in insect guts, play roles in nutrient absorption and pesticide degradation [85]. *Malassezia* species have been reported in lepidopteran insects [42], but their function remains unknown. Despite their ecological significance, fungi have often been overlooked in studies on gut microbes. However, they may contribute to nutrient supply, food breakdown and detoxification [46, 47]. Further research on fungal communities in herbivorous insects could reveal important interactions affecting host health and adaptability.

### Study Limitations

Our results should be interpreted with caution due to several limitations. We measured the microbiome only in the regurgitant, which originates from the foregut of caterpillars. This region is known to host a microbial community distinct from that of the midgut and to be more susceptible to external disturbances [29]. Therefore, we cannot exclude the possibility that a core microbiome may be present in the midgut. Although precautions were taken to avoid contamination during the collection of regurgitant samples, DNA extraction controls were not included in the analysis. As a result, the presence of potential contaminants cannot be entirely ruled out. The *Spodoptera* caterpillars used in this study were reared in the laboratory, which is known to significantly reduce gut microbiome diversity [42, 86]. In addition, the caterpillar species were not reared for the same number of generations: *S. frugiperda* and *S. latifascia* were reared for fewer than 10 generations, whereas *S. littoralis* and *S. exigua*, obtained from a commercial supplier, were likely maintained for more generations. This difference may explain some of the variation observed between these species. Finally, the identification of a core microbiome was based solely on occupancy, which may overlook rare microbial taxa with low detection probabilities, even though we used pooled samples from several individuals [87].

## Conclusion

In conclusion, this study compares gut bacterial and fungal communities across several *Spodoptera* species, including *S. latifascia*, whose microbiome had not been previously characterized. *S. latifascia* has a broad host range and may expand its distribution with climate change, increasing its pest potential. We found no evidence of a shared core gut microbiome, though laboratory rearing over multiple generations may have influenced this result. Although all species are highly polyphagous, none could feed on all tested crops, possibly due to differences in their bacterial gut communities. Characterizing these microbiomes can potentially support microbiome-based strategies for sustainable pest management [25].

## Supplementary Information

Below is the link to the electronic supplementary material.

Supplementary file1 (XLSX 1857 KB)

## Data Availability

The bacterial and fungal ASV count data per sample are available in the supplementary material (Tables S2 and S3). The sequencing data have been deposited in the NCBI Sequence Read Archive under BioProject accession number PRJNA1265134.
